# Modifying the five-time sit-to-stand test to allow use of the upper limbs: Assessing initial evidence of construct validity among lower limb prosthesis users

**DOI:** 10.1371/journal.pone.0279543

**Published:** 2023-02-10

**Authors:** Ignacio A. Gaunaurd, Sara J. Morgan, Geoffrey S. Balkman, Anat Kristal, Rachael E. Rosen, Jessica S. Haynes, Robert S. Gailey, Brian J. Hafner

**Affiliations:** 1 Department of Physical Therapy, University of Miami Miller School of Medicine, Miami, FL, United States of America; 2 Department of Rehabilitation Medicine, University of Washington School of Medicine, Seattle, WA, United States of America; 3 Spine Research Program, Gillette Children’s Specialty Healthcare, Saint Paul, MN, United States of America; 4 Research Division, The Geneva Foundation, Tacoma, WA, United States of America; University of Illinois at Chicago, UNITED STATES

## Abstract

The Five-time Sit-to-Stand (5xSTS) Test is a performance-based measure used by clinicians and researchers to assess the body functions needed to accomplish sit-to-stand transitions (e.g., lower limb strength, balance, and trunk control). The current requirements for performance of the 5xSTS Test (i.e., crossing arms over the chest) may not be appropriate for many, if not most lower limb prosthesis (LLP) users. The study aims were to (1) develop a modified five-time sit-to-stand (m5xSTS) Test protocol; (2) to examine initial evidence of known-groups construct validity among LLP users by comparing differences in performance by amputation level, amputation etiology, and functional level; and (3) to assess initial evidence of convergent construct validity by examining the correlations between m5xSTS performance with self-reported mobility (Prosthetic Limb Users Survey of Mobility (PLUS-M)), self-reported balance confidence (Activities-balance Confidence Scale (ABC)) and functional capability (comfortable walking speed). Three-hundred sixty-one LLP users participated in this cross-sectional study. The investigators developed a m5xSTS Test protocol that allows tested individuals to use different assistance strategies (i.e., use of upper limbs to push off thighs, push up from the armrests, or use a walker) when needed to perform the test. The investigators recorded m5xSTS Test times and assistance strategies. Significant differences in m5xSTS Test times were found between those who did and did not use an assistance strategy, as well as between participants grouped by different amputation level, etiology, and functional level. Significant moderate negative correlations were found between m5xSTS Test times and PLUS-M T-score (ρ = -0.42, *p*<0.001), ABC score (ρ = -0.42, *p*<0.001), and comfortable walking speed (ρ = -0.64, *p*<0.001), respectively. The m5xSTS Test allows LLP users to perform sit-to-stand transitions in a manner that accounts for their functional impairments, is consistent with post-amputation training, and is safe for the tested individual. Results from this study provide preliminary evidence of known groups and convergent construct validity for the m5xSTS Test with a large national sample of LLP users.

## Introduction

Standardized performance tests often require the tested individual to perform tasks in a specified manner (e.g., stand up from a chair with arms crossed across the chest). However, when assessing the functional capabilities of people with physical impairments, like lower limb prosthesis (LLP) users, administrators may want to allow the tested individual to use common assistance strategies when needed to better understand the person’s current capabilities when performing everyday activities. Understanding how an individual performs everyday activities using necessary assistance strategies can help clinicians develop patient-specific interventions best suited to the individual’s capabilities.

Sit-to-stand (and stand-to-sit) transitions are functional activities that LLP users perform frequently throughout the day. Bussman *et al*. reported that, compared to controls without amputation, LLP users had significantly lower overall physical activity but performed an equal number (i.e., 30–60) of daily sit-to-stand transitions [1]. The investigators suggested that sit-to-stand transitions may be unavoidable, even when the user’s activity is limited [1]. The ability to perform sit-to-stand transitions, with or without assistance strategies, may be important indicators of basic home and community mobility.

The Five-time Sit-to-Stand (5xSTS) Test is a performance test that was developed to measure elderly individuals’ physical functioning in both clinical care and research settings [2]. The 5xSTS Test was derived from the Timed-Stands Test which was previously developed by Csuka and McCarty as a means to measure muscle strength in people with lower limb weakness [3]. For the 5xSTS Test, Guralnik *et al*. reduced the number of sit-to-stand and stand-to-sit transitions (from 10 to 5) and required tested individuals to fold their arms across their chest to ensure they were not used during testing [2]. Researchers have subsequently used the 5xSTS Test to assess not only physical functioning but also other aspects of mobility like static and dynamic balance, trunk control, and dynamic postural stability [2–7].

Investigators who have previously studied the 5xSTS Test have reported that it has excellent test-retest reliability in multiple populations, including healthy active adults without current impairments, U.S. military service members, and adults with Parkinson’s disease, chronic obstructive pulmonary disease, and stroke [8,9]. While researchers have not yet evaluated the reliability and validity of the 5xSTS Test in LLP users, it has been used to assess mobility of LLP users (i.e., service members with unilateral transtibial amputation) relative to both healthy and injured (i.e., limb salvage) service members [10]. Researchers have also used the 5xSTS Test to examine the influence of different dynamic response feet on mobility in highly-functional LLP users [11], and to differentiate between LLP users with discrete levels of functional mobility [12].

Consistent with the developers’ intentions, investigators in prior studies of LLP users required participants to cross their arms over their chest during the 5xSTS Test [10–12]. However, maintaining one’s arms crossed over the chest during the 5xSTS Test requires a degree of lower limb strength, balance, and trunk control that many LLP users may not possess due to absence of or weakness in the lower limb muscles often used for sit-to-stand transitions (e.g., rectus femoris, hamstrings, tibialis anterior, tricep surae) [6,13]. To address these physical and functional impairments during rehabilitation, physical therapists teach LLP users assistance strategies they can use to perform sit-to-stand transitions. These strategies are intended to overcome the individual’s physical impairments with the intent of reducing effort and maximizing function [7,14]. For example, therapists teach LLP users to use organizational planning when rising from a chair [15]. The LLP user is instructed to: 1) move their buttocks forward such that their back is not supported by the chair placing their center of mass closer to their base of support; 2) place both feet flat on the floor with a comfortable width of support to promote symmetrical use of both lower limb; 3) flex their trunk forward during pre-ascent in an effort to use their momentum to assist with ascent initiation and seat off; and 4) use both upper limbs to press on the arm rests of the chair, utilizing the support of a walker, if needed, to complete and maintain quiet standing [16,17]. Even established LLP users utilize different assistance strategies (e.g., use of upper limbs to press on the thighs, press on the arm rests, use assistive devices, or combinations of these strategies) to perform daily sit-to-stand transitions [16,18–23]. Thus, the current requirements of the 5xSTS Test (i.e., crossing arms over the chest) may not be appropriate for many, if not most LLP users.

To address this limitation, we developed an alternative to the 5xSTS Test, termed the modified Five-Time Sit-To-Stand (m5xSTS) Test, which allows LLP users to perform the test while using their upper limbs. However, the validity of the m5xSTS Test must be established before it can be recommended for use in clinical practice or research. Therefore, the goals of this study were to assess the construct (i.e., convergent and known groups) validity of m5xSTS Test in a large national sample of LLP users. We hypothesized that significantly lower m5xSTS Test times (i.e., better performance) would be observed in LLP users who did not use an assistive strategy, as well as those who had more distal amputations (i.e., below-the-knee better than above-the-knee), different causes of amputation (i.e., trauma, tumor, congenital, and other better than dysvascular and infection), and higher functional levels (i.e., Medicare Functional Classification Level K4 better than K3; K3 better than K2; K2 better than K1). Further, we hypothesized that m5xSTS Test times would correlate moderately and significantly with comfortable walking speed (i.e., a similar measure of mobility performance) and moderately and significantly with self-reported mobility and balance confidence (i.e., similar constructs measured using standardized survey instruments). Confirmation of these hypotheses would serve as initial evidence of known groups and convergent construct validity for the m5xSTS Test, as well provide data that could serve as reference values for this test.

## Materials and methods

This study was part of a cross-sectional study of LLP users conducted between October 2018 and February 2020. We recruited participants from prosthetics clinics, rehabilitation hospitals, and limb loss support groups in 17 cities across North America. Data were collected in-person by the investigators at indoor testing locations that included community centers, classrooms, laboratories, hospitals, prosthetics clinics, and conference halls. A University of Washington Review Board reviewed and approved study procedures. All participants provided written informed consent prior to testing.

### Participants

We targeted a sample of 500 participants so as to establish reference values for large, national sample of LLP users. We required participants to be at least 18 years of age, be able to read English, have a unilateral lower limb amputation at or between the ankle and hip joints, be able to ambulate independently with a prosthesis, and have at least three months experience using a prosthesis. We excluded participants if they had a contralateral lower limb amputation, an upper limb amputation, or presented at the time of testing with a health condition (e.g., history of stroke) other than lower limb amputation that affected their balance or ability to walk. We asked participants to wear comfortable clothing and footwear they typically wore with their current prosthesis.

### Study procedures

A study investigator interviewed participants to determine level and cause of amputation. We categorized the participant’s level of amputation as either below-the-knee, which consisted of those with an ankle disarticulation (AD) or a transtibial amputation (TTA), or above-the-knee, which consisted of those with a knee disarticulation (KD), transfemoral amputation (TFA), or hip disarticulation (HD). We classified cause of amputation as dysvascular, trauma, infection, tumor, congenital limb absence/difference, or other (e.g., failed limb salvage). A licensed certified prosthetist assigned each participant’s Medicare Functional Classification Level (MFCL, “K-level”) after discussing with the participant their typical daily and recreational activities, inspecting the participant’s prosthesis, and observing the participant ambulate. Participants then completed a short survey that included demographic questions, as well as questions about the participant’s health (i.e., presence of comorbidities included in the self-report version of the Charlson Comorbidity Index [24]), amputation-related medical history, and prosthesis use. The survey also included the Prosthetic Limb Users Survey of Mobility (PLUS-M) and Activities Balance Confidence (ABC) Scale which are measures of self-perceived prosthetic mobility and balance confidence, respectively [25,26]. Higher T-scores on the PLUS-M and scores on the ABC (0–4 scale) indicate greater self-perceived prosthetic mobility and balance confidence, respectively. We selected these instruments as they measure similar constructs (i.e., balance confidence and prosthetic mobility) to the 5xSTS Test. Other investigators have used the ABC previously to assess evidence of criterion validity of the 5xSTS Test in people with balance disorders [5]. Once participants completed the survey, investigators administered a modified version of the 5xSTS Test.

### Modified five-time sit-to-stand (m5xSTS) test

We developed a modified Five-Time Sit-to-Stand (m5xSTS) Test to allow participants with lower limb amputation use of their upper limbs. The m5xSTS Test protocol (S1 Appendix) included explicit participant instructions based on a review of pertinent literature [2–5,10]. We administered m5xSTS Test using a standard height office reception chair with bilateral armrests (Devoko Office Guest Chair, height: 44.5cm, seat depth: 45cm, armrest height: 64cm). An investigator who was either a licensed physical therapist or a licensed and certified prosthetist administered the m5xSTS Test. The administrator first provided participants with the specified test instructions, and then demonstrated how to stand and sit five times. All participants were fit with a gait belt for safety during testing. The participant began the test seated, and a walker (Medline G07767) was placed in front of the participant if needed for assistance. The administrator used a stopwatch (Accusplit A601X, Pleasanton, CA) to measure time while simultaneously observing the assistance strategy used by the participant to perform the test. The time required to complete the test (to 1/10^th^ of a second) and assistance strategy (use of the thighs, armrests, and/or walker) used during any of the sit-to-stand repetitions were documented.

### Comfortable walking speed test

After completing the m5xSTS Test, participants performed a walking test to determine their comfortable walking speed. Researchers often use walking speed to establish evidence of criterion and construct validity for performance tests of mobility. For example, investigators previously used walking speed to assess the criterion validity of the 5xSTS Test in adults with balance impairments [27]. As with the m5xSTS Test, we developed a standardized protocol to administer the walking test. Participants were asked to walk a distance of 5m, turn around a cone in their preferred direction, and return 5m to the starting line (i.e., total of 10m). We developed this walking test protocol as a practical means to assess LLP users across a greater range of settings where we expected to administer the m5xSTS Test. A recent survey of prosthetists in the U.S. found that only 31% of respondents had space for a 10-meter walk test; however, 93% of respondents had a 5-meter space where patients could walk and turn around [28].

The same investigator administered the comfortable speed walking test and the m5xSTS Test. The same stopwatch was used to measure the time required to complete the task (to 1/10^th^ of a second). Walking test instructions were:

*“The goal of this test is to walk around the cone and back at your usual pace. Stand with your toes behind the lines. When I say ‘go,’ walk forward and around the cone in either direction. Walk back and stop when you cross the line. Use your assistive device if you need at any time. Move safely, efficiently, and at a comfortable speed. Are you ready? 3…2…1…go”*.

Comfortable walking speed (m/s) was determined by dividing the total distance (10m) by the time required to complete the task. The task was repeated if the participant walked past the starting/finish line without slowing down and/or the cone was kicked out of position.

### Data analyses

We performed all statistical analyses using SPSS Statistics 26 (IBM Corporation, Armonk, NY). We used descriptive analyses to characterize the study population as well as to describe the frequency of assistance strategies used by participants to perform the m5xSTS Test. The Shapiro Wilk test demonstrated that the m5xSTS Test times were not normally distributed (p<0.001). We used the Mann Whitney U Test to assess differences in m5xSTS Test performance between participant subgroups defined by gender (male/female), level of amputation (below-knee/above-knee), and use of an assistance strategy (no assistance/assistance). We used the Kruskal-Wallis H Test to assess differences in m5xSTS Test performance across participant subgroups defined by assistance strategy (no strategy, use of thighs only, use of arm rests only, and use of 2 or more strategies); amputation etiology (dysvascular, trauma, infection, tumor, congenital, and other); and MFCL (K1, K2, K3, and K4).

Expectations based on previously published literature describing known group differences in performance tests and self-report measures of mobility suggested that m5xSTS Test performance would differ based on amputation level (below knee vs above knee) and assistance strategy (no strategy vs one or more strategies) because of the differences in remaining intact anatomical and musculature tissues between the groups.[29] We also expected differences in m5xSTS Test performance based on MFCL, which is a standard rating of functional mobility in LLP users. Investigators have used MFCL previously to establish known groups based on mobility in numerous studies [25,30,31].

We calculated the Spearman correlation coefficient (ρ) to determine the strength and relationship between the m5xSTS Test, and scores from instruments selected to assess convergent validity (i.e., PLUS-M, ABC, and comfortable walking speed). We considered correlation coefficients of 0.1 to 0.3, 0.4 to 0.6, and 0.7 to 0.9 to be weak, moderate, and strong, respectively [32]. From previous research on the 5xSTS Test, we expected to observe a moderate correlation between m5xSTS Test times and comfortable walking speed, and a moderate correlation between m5xSTS Test times and survey scores (i.e., PLUS-M T-scores and ABC scores) [5,25,27].

## Results and discussion

### Participant characteristics

We administered the m5xSTS Test to 362 LLP users with unilateral amputation before suspending data collection due to the COVID-19 pandemic (Table 1). Of the 362, only one participant was unable to perform the test therefore their data were excluded from the validity analysis. More participants had a below-the-knee amputation (65%) than an above-the-knee amputation (35%). Most participants (94%) had a mid-segment amputation (i.e., TTA or TFA); fewer (6%) had a through-joint amputation (i.e., AD, KD, or HD). About half of the participants had an amputation due to trauma (50%), followed by dysvascular disease (19%), and infection (14%). We classified most participants (86%) as unlimited community ambulators (i.e., MFCL K3 or K4), while the remainder (14%) were deemed to be limited community or household ambulators (i.e., MFCL K1 or K2) [33].

**Table 1:** Characteristics of the study sample (N = 362).

Characteristic	Median	Range
Age (years)	55.0	18.4–84.9
Height (cm)	175.3	149.9–195.6
Weight (kg)	83.9	44–177
Time since amputation (years)	8.5	0.2–72.2
**Characteristic**	**n**	**%**
Gender Male Female	244 118	67 33
Race/ethnicity American Indian / Alaskan Native Asian Black or African American Hispanic Native Hawaiian or other Pacific Islander White or Caucasian More than 1 Race Unknown or not reported Amputation Level AD TT KD TF HD	3 10 41 41 1 251 13 2 5 232 15 107 3	1 3 11 11 <1 69 4 1 1 64 4 30 1
Amputation etiology Trauma Dysvascular Infection Tumor Congenital Other	182 67 50 34 20 9	50 19 14 9 6 2
MFCL K1 K2 K3 K4	4 48 171 139	1 13 47 39

Abbreviations: AD = ankle disarticulation; TTA = transtibial amputation; KD = knee disarticulation; TFA = transfemoral amputation; HD = hip disarticulation; MFCL, Medicare Functional Classification Level.

In terms of health, 51% of participants reported no co-morbidities, 26% reported one co-morbidity, 13% reported two co-morbidities, and 10% reported three or more co-morbidities. Arthritis and diabetes were the most reported co-morbidities. Of those participants who reported having diabetes, 38% reported no other co-morbidities, 26% reported having one additional co-morbidity, and 37% reported having two or more additional co-morbidities.

### m5xSTS test results

While all but one participant (i.e., a 47-year-old K3 ambulator with transtibial amputation due to dysvascular disease) were able to perform the m5xSTS Test, almost two-thirds of participants (n = 229) performed the m5xSTS Test using an assistance strategy (Table 2). Among those who used assistance strategy, the most common single strategy used was the chair armrests, followed by thighs, and the walker (about 69%, 20%, and < 1% of the sample, respectively). About 11% of participants who used assistance strategy, used two or more strategies. Armrests were frequently used by participants who had a more proximal amputation level or had an amputation due to vascular causes. Most participants (79%) who had an above-the-knee amputation and were classified as MFCL K1-K3 used just the armrests to perform the test. Fewer than 15% of all participants used just their thighs to perform the test. Only two participants used the walker alone to perform the m5xSTS Test. Both participants were classified as K2 ambulators, lost their limb due to dysvascular reasons or infection, had either a TTA or KD, and performed the m5xSTS Test in 16.7 s or 18.5 s, respectively. An additional 22 participants used the walker in combination with another assistance strategy to perform the m5xSTS Test. A total of 26 participants used two or more types of assistance strategies. Of them, 21 used armrests and the walker, four used their thighs and the armrests, and one used all three assistance strategies.

**Table 2:** Assistance strategies used by participants (n = 361) to complete the m5xSTS Test based on amputation level, cause of amputation, age, and Medicare Functional Classification Level (MFCL).

Category	Without assistance strategy		With assistance strategy		Specific assistance strategies							
					Thighs		Armrests		Walker		≥2 strategies	
	n	%*	n	%*	n	%*	n	%*	n	%*	n	%*
**Amputation Level**												
Below-the-knee (AD, TTA)	99	42	137	58	39	16	82	35	1	<1	15	6
Above-the-knee (KD, TFA, HD)	33	26	92	73	6	5	75	60	1	<1	10	8
**Amputation etiology**												
Trauma	76	42	106	58	29	16	69	38	0	0	8	4
Dysvascular	10	15	56	85	9	14	37	56	1	<1	9	14
Infection	11	22	39	78	3	6	28	56	1	2	7	14
Tumor	13	38	21	62	2	6	19	56	0	0	0	0
Congenital	16	80	4	20	1	5	2	10	0	0	1	5
Other	6	67	3	33	1	11	2	22	0	0	0	0
**MFCL**												
K1	0	0	4	100	0	0	2	50	0	0	2	50
K2	2	4	46	96	2	4	33	69	2	4	9	19
K3	43	25	127	75	22	13	93	55	0	0	12	7
K4	87	63	52	37	21	15	29	21	0	0	2	1

* The percent of participants in the category who used the specified assistance strategy (e.g., the number of participants with below knee amputation who used no strategy). Abbreviations: AD = ankle disarticulation; TTA = transtibial amputation; KD = knee disarticulation; TFA = transfemoral amputation; HD = hip disarticulation; MFCL, Medicare Functional Classification Level.

The median (range) m5xSTS Test time (seconds) for the sample was 13.1 s (6.0–54.3 s). The 75th and 25th percentiles were 10.8 s and 16.8 s, respectively. The median (range) m5xSTS Test times for those who did and did not use an assistance strategy was 15.2 s (7.8–54.3 s) and 11.1 s (6.0–20.8 s), respectively. Correlations between m5xSTS Test times and participants’ anthropometric measurements such as body weight (ρ = 0.28, *p*<0.001) and height (ρ = 0.17, *p*<0.001) were weak.

There were significant differences in m5xSTS Test times between those who did and did not use an assistance strategy (*U* = 5913, *z* = -9.64, *p*<0.001). There were also significant differences in test times between assistance strategy subgroups (*H*(6) = 121.06, *p*<0.001). The median (range) m5xSTS Test times for males and females were 13.5 s (6.0–45.1 s) and 12.4 s (6.15–54.3 s), respectively. However, there were no significant differences in test times between genders (*U* = 13613, *z* = -0.78, *p* = 0.44). For participants with below-knee amputation (i.e., AD and TTA), there were significant differences in m5xSTS Test times among all assistance strategy subgroups when compared to the no assistance strategy subgroup (*H*(3) = 67.48, *p*<0.001). For participants with above-knee amputation (i.e., KD, TFA, and HD), there were significant differences in m5xSTS Test times between the no assistance subgroup and the subgroups who used the armrest or two or more types of assistance strategies (*p*<0.001) (Table 3).

**Table 3:** The m5xSTS test times (seconds) by level of amputation and use of assistance strategy (n = 359^a^). Median and quartiles are provided for each subgroup in the following format: Median (75^th^ quartile;25^th^ quartile), minimum-maximum, and subgroup sample size. ^a^The two participants who used the walker as their only assistance strategy were not included in these analyses.

Level of Amputation	No Assistance Strategy (None)	Use of Thighs (Thighs)	Use of Armrests (Armrests)	≥2 Assistance Strategies (Two)	Significant differences between subgroups
Below knee (AD, TTA)	10.7 (9.1;12.3) 6.0–20.8 n = 99	11.5 (10.3;14.8) 8.2–22.8 n = 40	15.3 (13.0;17.6) 8.6–29.9 n = 81	18.7 (14.5;26.9) 8.9–48.7 n = 15	None-Armrests***; None-Thighs*; None-Two***; Thighs-Armrests***; Thighs-Two***; Armrests-Two*
Above knee (KD, TFA, HD)	11.4 (10.3;14.8) 7.2–18.3 n = 33	14.0 (13.3;15.3) 10.4–18.6 n = 6	16.0 (13.1;19.1) 7.8–32.6 n = 74	20.3 (15.1;27.5) 10.8–54.3 n = 11	None-Armrests***; None-Two***
All levels	11.1 (9.3;12.5) 6.0–20.8 n = 132	11.7 (10.4;15.3) 8.2–22.8 n = 46	15.4 (13.1;18.6) 7.8–32.6 n = 155	19.5 (14.5;26.9) 8.9–54.3 n = 26	None-Armrests***; None-Thighs*; None-Two***; Thighs-Armrests***; Thigh-Two***; Armrests-Two*

* = *p*<0.05

*** = *p*<0.001.

Abbreviations: AD = Ankle Disarticulation; TTA = Transtibial Amputation; KD = Knee Disarticulation; TFA = Transfemoral Amputation; HD = Hip Disarticulation.

In general, there were significant differences in m5xSTS Test times between participants with below-the-knee and above-the-knee amputation levels (*U* = 18866, *z* = 4.36, *p*<0.001). There were also significant differences in m5xSTS Test times between those with below-the-knee and above-knee amputation levels who did not use assistance (*U* = 2034, *z* = 2.11, *p* = 0.035). There were also significant differences in m5xSTS Test times between the amputation levels for those who used assistance (*U* = 7511 *z* = 2.46, *p* = 0.014).

Test times were also significantly different among participants with different causes of amputation (*H*(5) = 52.58, *p*<0.001)). Specifically, participants with dysvascular amputation were often significantly slower than participants with other causes of amputation (Table 4). For those who did not require assistance, significant differences in m5xSTS Test times were found between those with a cause of amputation of trauma, tumor, congenital or other and those with a cause of amputation of dysvascular or infection (*U* = 796, *z* = -2.23, *p* = 0.022). For those participants who required assistance, significant differences in m5xSTS Test times were found between those with a cause of amputation of trauma, tumor, congenital or other and those with a cause of dysvascular or infection (*U* = 4299, *z* = -4.18, *p*<0.001).

**Table 4:** The m5xSTS test times (seconds) by level and cause of amputation (n = 361). Median and quartiles are provided for each subgroup in the following format: Median (75th quartile;25th quartile), minimum-maximum, and subgroup sample size.

Level of Amputation	Dysvascular (Dys)	Trauma (Tr)	Infection (Inf)	Tumor (Tum)	Congenital (Con)	Other (Oth)	All causes	Significant differences between subgroups
Below knee (AD, TTA)	16.1 (13.5;18.8) 10.1–48.7 n = 51	11.7 (9.5;14.0) 6.1–34.5 n = 116	13.9 (12.0;17.3) 8.6–26.3 n = 36	10.5 (9.2;11.4) 7.2–13.3 n = 12	10.5 (9.5;12.4) 6.0–16.7 n = 16	10.2 (10.0;11.3) 9.7–12.2 n = 5	12.4 (10.3;15.9) 6.0–48.7 n = 236	Dys-Tr***;Dys-Tum***;Dys-Con***; Dys-Inf*; Dys-Oth***;Inf-Tum***;Inf-Con**;Inf-Tr***; Inf-Oth*
Above knee (KD,TFA,HD)	18.6 (16.9;24.2) 14.3–54.3 n = 15	13.8 (11.5;17.7) 7.2–50.7 n = 66	13.1 (11.9;17.4) 10.8–29.2 n = 14	15.1 (10.6;18.1) 8.9–29.3 n = 22	15.6 (13.8;18.0) 11.0–22.8 n = 4	14.2 (13.3;14.7) 11.1–16.1 n = 4	14.5 (11.8;18.5) 7.2–54.3 n = 125	Dys-Tr**;Dys-Tum**; Dys-Inf*
All levels	16.6 (13.8;19.8) 10.1–54.3 n = 66	12.5 (10.3;15.8) 6.1–50.7 n = 182	13.6 (12.0;17.5) 8.6–29.2 n = 50	11.5 (10.2;16.9) 7.2–29.3 n = 34	11.0 (9.9;14.2) 6.0–22.8 n = 20	11.3 (10.2;14.1) 9.7–16.1 n = 9	13.1 (10.8;16.7) 6.0–54.3 n = 361	Dys-Tr***;Dys-Tum***;Dys-Con***; Dys-Inf**;Dys-Oth***;Inf-Con**; Tr-Inf*

* = *p*<0.05

** = *p*<0.01

*** = *p*<0.001.

Abbreviations: AD = Ankle Disarticulation; TTA = Transtibial Amputation; KD = Knee Disarticulation; TFA = Transfemoral Amputation; HD = Hip Disarticulation.

We also observed that m5xSTS Test times were significantly different between MFCL subgroups (*H*(3) = 100.10 *p*<0.001)), with higher MFCL subgroups generally exhibiting faster times. Similarly, there were significantly faster m5xSTS Test times for participants with higher MFCL compared to participants with lower MFCL within the amputation level groups (Table 5).

**Table 5:** The m5xSTS test times (seconds) by Medicare Functional Classification Level (K-level) and level of amputation (n = 361). Median and quartiles are provided for each subgroup in the following format: Median (75th quartile;25th quartile), minimum-maximum, and subgroup sample size.

Level of Amputation	K1	K2	K3	K4	Significant differences between subgroups
Below knee (AD, TTA)	22.1^§^ n = 1	17.0 (14.3;20.2) 8.6–48.7 n = 32	13.5 (11.5;16.6) 7.5–29.9 n = 106	10.7 (9.1;12.0) 6.0–20.8 n = 97	K2-K3***; K2-K4***; K3-K4***
Above knee (KD, TFA, HD)	18.0 (17.0;36.2) 15.9–54.3 n = 3	19.0 (16.3;29.0) 10.8–32.6 n = 16	15.7 (13.1;18.4) 7.8–50.7 n = 64	12.3 (10.5;15.4) 7.2–21.9 n = 42	K2-K3*; K2-K4***; K3-K4*** K1-K4*
All Levels	20.1 (17.5;30.2) 15.9–54.3 n = 4^§^	17.6 (14.3;22.5) 8.6–48.7 n = 48	14.3 (12.0;17.3) 7.5–50.7 n = 170	11.1 (9.3;13.0) 6.0–21.9 n = 139	K1-K3*; K2-K3***; K2-K4***; K3-K4***; K1-K4***

* = *p*<0.05

*** = *p*<0.001

§ = Not included in the below-knee-amputation statistical analysis.

Abbreviations: AD = Ankle Disarticulation; TTA = Transtibial Amputation; KD = Knee Disarticulation; TFA = Transfemoral Amputation; HD = Hip Disarticulation.

The median (range) comfortable walking speed for this sample was 0.96 m/s (0.10–1.70 m/s) which falls within the range of a community ambulator [34]. The median (range) for PLUS-M T-score was 57.3 (32.2–71.4) which is at the 76.7 percentile of self-perceived prosthetic mobility. The median (range) for ABC score was 3.3 (0–4.0), which is between high balance confidence and complete balance confidence when performing different daily activities. There was a moderate negative statistically significant correlation between m5xSTS Test times and PLUS-M T-score (ρ = -0.42, *p*<0.001) and ABC (ρ = -0.42, *p*<0.001) score, respectively. There was also a moderate negative statistically significant correlation between m5xSTS Test times and comfortable walking speeds (ρ = -0.64, *p*<0.001). These correlations indicate that better m5xSTS Test performance was associated with greater self-perceived mobility and balance confidence, and faster comfortable walking speed.

## Discussion

For this study, we developed a protocol (S1 Appendix) for a modified version of the 5xSTS Test. Over the last 26 years, investigators have administered the 5xSTS Test differently than how it was first proposed by Guralnik *et al*. [2] In a meta-analysis of 5xSTS Test reference values for the geriatric population [4], the author noted inconsistencies in how the 5xSTS Test has been administered, including: 1) beginning the test on movement initiation or on “go”; 2) ending test in standing or sitting position; and/or 3) specifying the position of arms (i.e., arms folded across the chest or not). Of note, the author reported that two of the 14 studies allowed participants to use their upper limbs if they were unable to perform the 5xSTS Test with their arms folded across their chest. In the present study, we modified the 5xSTS Test protocol to allow participants (i.e., LLP users), who may lack the capability to stand without using arms, to use them if needed.

To assess the validity of this m5xSTS Test, we also administered the test to a large, diverse national sample of LLP users. The study sample was similar, both in demographic characteristics (e.g., age, gender, race, ethnicity) and amputation-related characteristics (e.g., level and etiology of amputation), to recent, large studies of LLP users in the United States [35,36]. However, we had more participants classified as MFCL K4 (40%) and fewer participants classified as MFCL K2 (14%) than are typically seen in routine prosthetics practices (i.e., 9–10% and 36–37%, respectively) [37,38].

Results from this study confirmed our hypotheses that m5xSTS Test times differ significantly across groups defined by type of assistance strategy selected, amputation level, amputation etiology, and functional level. For example, m5xSTS Test times were significantly slower for LLP users who relied upon upper limb support to complete the test (Table 3). As expected, test performance decreased with increasingly supportive assistance strategies. A common clinical goal is to improve mobility through independence and efficiency with transitional movements such as sit-to-stand. For example, in rehabilitation, a LLP user may progress from needing multiple types of assistance (e.g., pushing through the armrests of the chair and using a standard walker) to use of a single type of assistance (e.g., pushing through the thighs) to no assistance strategy. Pushing through the thighs is a clinical progression between the use of armrests and no assistance strategy. This strategy facilitates upward momentum and assists with shifting the center of mass over the base of support [15]. Further, pushing on the thighs could provide proprioception, stabilization, and greater vertical force through the lower limbs during a transitional movement that is often significantly asymmetrical in people with lower limb amputation [15–17]. Therefore, testing sit-to-stand ability while allowing the LLP users to use the assistance strategy (or strategies) they would use if they were unable to perform the test as instructed may have greater clinical relevance and utility for this population than testing without use of arms (e.g., as per protocol with the 5xSTS Test) [2].

Results of the present study also demonstrated that participants with dysvascular amputation etiology, lower functional level, and more proximal amputation level expectedly performed the test slower than LLP users with other amputation etiologies, higher functional levels, and more distal amputation levels. These findings are similar to previous 5xSTS Test studies that found differences in 5xSTS Test performance based on functional level (e.g., MFCL K3 vs K4) [12]. These data also suggest that the m5xSTS Test may be able to discriminate between groups as well as the original version, but is likely to be applicable to a greater range of LLP users (i.e., people classified as MFCL K1 or K2). Also, because we recruited such a large, national study sample, data in Tables 3–5 can also serve as reference values for the m5xSTS Test and provide clinicians and researchers with information to evaluate an individual’s performance in context of their peers. Data from this study can be used to benchmark an individual patient’s m5xSTS Test performance relative to LLP users with the same amputation level, amputation etiology, and MFCL level.

Lastly, we were able to establish initial evidence of convergent construct validity of the m5xSTS Test by demonstrating moderate and significant negative relationships with measures of self-perceived mobility (PLUS-M) and balance confidence, which is consistent with previously published validity testing literature between self-report surveys and performance tests in LLP users [25,39,40]. In recent years, the walking speed has been proposed as a functional vital sign along with the six traditional vital signs (blood pressure, heart rate, temperature, respiration rate, height, and weight) that assess patient’s physiological function [34]. The significant moderate negative relationships found between m5xSTS Test and comfortable walking speed could potentially inform the clinician not only on lower strength, balance, and trunk control but also on the functional and aerobic capability of the LLP user.

### Study limitations

Although we found that LLP users in this study opted to use different assistance strategies when they were unable to perform the m5xSTS Test as instructed, we did not collect data on whether they could perform the test with an assistance strategy other than the one(s) they selected. We also do not know whether the assistance strategies selected were related to lack of lower limb strength, impaired balance, limited lower limb power, previous training, and/or habitual tendencies. Because MFCL K4 represents the highest level of functional potential, one might assume that these individuals should be able to perform repeated sit-to-stand transitions without upper limb assistance (a supposition supported by 5xSTS Test results from Beisheim *et al*. [12]. However, 37% of participants in this study who were classified as K4 elected to use either the armrests or their thighs to perform the m5xSTS Test. Future studies could be done to examine how m5xSTS Test performance would be affected by requiring participants to use specific assistance strategies.

Another limitation of the present study is that, thus far, we have obtained only preliminary evidence of known groups and convergent construct validity of the m5xSTS Test in LLP users. Future work is needed to assess other important psychometric properties, such as test-retest reliability (including measurement error), and responsiveness of this test. Establishing evidence of these measurement properties will help to encourage use of the m5xSTS Test for applications other than discrimination (e.g., longitudinal evaluation of prosthetic patients).

While m5xSTS Test performance was only weakly correlated with weight and height, it is possible that test performance could be affected by other anthropometric or body composition measurements. Researchers have advocated previously for normalizing or scaling results from performance tests to mitigate effects of confounding variables like limb segment length, lean body mass, and body fat percentage on key aspects of physical functioning (e.g., muscle strength and power) [41]. If test performance is confounded by these variables, then it could affect interpretation of individual scores and comparison to reference values obtained from the present study. While recommendations proposed previously [41] may guide efforts to assess the need for normalization of the m5xSTS Test, additional work is needed to assess whether allometric models established previously for people with presumably intact limbs would apply to people with different levels of amputation and varying residual limb lengths. Additionally, rigorous study is needed to determine how well normalization strategies proposed for tests intended to measure performance variables like torque, force, velocity, and power would apply to tests like the m5xSTS Test that are intended to assess aspects of mobility like static and dynamic balance, trunk control, and dynamic postural stability.

Another limitation of this study is that we collected performance data from established LLP users. Thus, the results we obtained would not apply to other clinical populations. Additional work is needed to administer the m5xSTS Test to LLP users who are early post-amputation and just fitted with a prosthesis or undergoing changes in treatment (e.g., physical therapy or receiving a new prosthesis). Results of such research could be used by clinicians and researchers to optimize timing and duration of therapeutic or prosthetic treatments, reduce secondary co-morbidities, and improve quality of life in LLP users.

## Conclusion

We developed a modified version of the 5xSTS Test that allows LLP users to use their arms during testing if they are unable to perform the test as instructed. We then administered the m5xSTS Test to a large, national sample of LLP users to assess known groups and convergent construct validity. Results from testing showed that LLP users often use different assistance strategies when performing the m5xSTS Test, and that test times expectedly differed significantly across groups defined by amputation level, amputation etiology, and functional level. Results from the m5xSTS Test administered in this study may also serve as reference values for LLP users assessed in clinical practice or research, as we present m5xSTS Test times by groups of participants with different levels of amputation, etiologies of amputation, and functional levels.

## Supporting information

S1 Appendix Appendix 1: Modified five-time sit-to-stand (m5xSTS) test protocol. (DOCX)

S1 File (XLSX)
