# Supplementary material for: Modifying the five-time sit-to-stand test to allow use of the upper limbs: Assessing initial evidence of construct validity among lower limb prosthesis users
Source: PLoS One. 2023 Feb 10;18(2):e0279543. doi: 10.1371/journal.pone.0279543 (PMC9916626; doi:10.1371/journal.pone.0279543)
Supplement: S1 Appendix — (DOCX) [file pone.0279543.s001.docx]

**Appendix 1: Modified Five-Time Sit-to-Stand (m5xSTS) Test Protocol**

Equipment:

The m5xSTS test can be administered by one clinician; an assistant may be used if required for safety. A standard height office reception chair with bilateral armrests (approximate height: 44.5 cm; approximate depth: 45 cm; approximately armrest height: 64 cm), a stopwatch, a gait belt, and a standard or bariatric walker will be required for administration.

Set-up:

Place the chair in direct contact with a stationary vertical surface (e.g., wall or column) so that it does not move during the test (alternatively, test administrators can place a foot behind the chair leg to keep it from moving). The participant will begin the test seated in the chair. A standard or bariatric walker will be placed in front of the seated participant to provide assistance, if it is needed. Be sure to position the walker so that it is available if needed but will not interfere with testing if not required.

Administration:

Perform the test by providing the verbal instructions (in italics), demonstrating the test, and then administering the test to the participant.

“*The goal of this test is to stand up and sit down quickly five times. Sit forward in the chair with your back straight and your hands together (on your lap). When I say “go”, stand up straight and sit back down five times. Make sure your buttocks touch the chair each time. Use the armrests or walker if you need to at any time. I will now demonstrate.”*

Demonstrate to the participant how to stand and sit five times, while keeping your hands together in front of you. Ask the participant if they have any questions. Tell the participant to sit in the starting position, and begin the test by reading the final instructions.

“*Move safely, efficiently, and at a fast speed. Are you ready? 3….2….1….go”*.

Start the stopwatch when you say “go.” Count aloud each time the participant’s buttocks touches the seat of the chair. Stop the stopwatch when the participant’s buttocks touch the seat of the chair on the fifth count.

Retesting:

The participant is only required to perform the test once, but have them repeat the test if: 1) they did not stand up straight before sitting down on any repetition; 2) their buttocks did not come in contact with the chair; and/or 3) the chair moves out of position during the test. Give the participant a 60-second rest period prior to repeating the test if a retest is required.

Scoring:

Record the total time required to complete the test, as well as any use of the upper limbs to push on and/or stabilize against the thighs, armrests of the chair, and/or walker.

| Record m5xSTS time: | Record any assistance strategy used (check all that apply): |
| --- | --- |
| ____ : ____ . ____ seconds | No strategy used  Used knees  Used armrests  Used walker |
